# Menu Planning in Residential Aged Care—The Level of Choice and Quality of Planning of Meals Available to Residents

**DOI:** 10.3390/nu7095354

**Published:** 2015-09-09

**Authors:** Karen L. Abbey, Olivia R. L. Wright, Sandra Capra

**Affiliations:** Centre for Dietetics Research, School of Human Movement and Nutrition Sciences, The University of Queensland, St Lucia QLD 4072, Australia; E-Mails: o.wright@uq.edu.au (O.R.L.W.); s.capra@uq.edu.au (S.C.)

**Keywords:** menu planning, choice, quality, Aged Care Standards

## Abstract

Background: Choice of food is an imperative aspect of quality of life for residents in Residential Aged Care Homes (RACHs), where overall choice and control is diminished upon entering a home to receive care. The purpose of this study was to examine the current strategies of menu planning in a range of RACHs in Australia, and whether this facilitated appropriate levels of choice for residents receiving texture modified and general diets. Methods: The study comprised a National Menu Survey using a new survey instrument collecting general information about the RACH and foodservice system, menu information and staffing information (*n* = 247); a national menu analysis (*n* = 161) and an observational case study of 36 meal environments. Results: Choice was low for the entire sample, but particularly for those receiving pureed texture modified diets. Evidence of menu planning to facilitate the inclusion of choice and alternatives was limited. Discussion: Regulation and monitoring of the Australian Aged Care Accreditation Standards needs to be strengthened to mandate improvement of the choice and variety offered to residents, particularly those on pureed texture modified diets. Further research on how menu choice and a lack of variety in meals affects the quality of life residents is needed in this context, but current evidence suggests the effect would be detrimental and undermine resident autonomy and nutritional status.

## 1. Introduction

Providing food choice is very important in the aged care sector, where quality of life is paramount [1]. The actual food choices vary with individuals and include unconscious and conscious decisions made by an elderly person before or at the point of food consumption [2]. Food choice is a dynamic and complex process being affected by personal factors, the expectation of specific foods, appetite, mood, emotions, socio-economic factors—such as the meaning and status of food and income, as well as educational factors—such as knowledge about nutrition and food [3,4]. The intrinsic factors of food, such as appearance, odour, texture, colour, flavour, quantity, quality preparation, and presentation, also play a part in determining why people choose specific foods [2,5]. Being able to choose what one eats is a fundamental right of every resident living in Residential Aged Care Homes (RACHs) in Australia [6]. This is particularly important given the high rates of undernutrition of between 30%–65% in the long-term care sector internationally, which predisposes residents to a greater risk of illness and functional decline [1].

### 1.1. Autonomy in Food Choice

Residents in RACHs need to have the ability to exercise food choice in order to maintain some autonomy over their food environment. This has previously been demonstrated as a very important aspect of foodservice provision in RACHs [1]. The nature of admission to a RACH proves challenging for residents, as they are required to swiftly adjust to existing meal structures and systems. Specifically, the menu is already set and organised, as are meal times, meal choices, dining settings and dining companions [7]. In essence, residents experience imposed restrictions and controls almost immediately, thus putting their sense of autonomy at risk. Aside from the impact on autonomy, the menu provided in RACHs is the sole source of nutrition for residents. Providing a range of alternative meal choices is important to provide adequate nutrition and to encourage meal consumption [8]. People eat foods, not nutrients, and residents control their nutritional state by choosing the foods they like to eat. The menu therefore needs to offer a sufficient variety of foods across the day to suit residents’ preferences [9], as well as provide a realistic choice.

Increased levels of foodservice satisfaction of up to 30% have occurred when residents have the power to choose from the menu [10]. It was also found when asked to rate the importance of control and choice over certain areas of their everyday life in a home, residents prioritised having choice over their foods as the most important [10,11]. This increases both pleasure and enjoyment, which in turn can impact quality of life [6,12]. Increased independence in food choice and active participation in food provision has been linked to reduced nutritional risk [13]. Choice also increases the motivation for residents to better consume their meals [10,14,15,16]. Despite these positives, it is often found that autonomy remains severely limited in the aged care setting. The issue is not limited to Australia, as in a study of 17 homes in the United States of America (USA), 58% of residents reported having no choice about menu selection [17].

### 1.2. Timing of Meal Choice

The meal environment is defined as the system that underpins the menu, meal production and meal delivery. It needs to support food choice in terms of the timing when residents can select food items from the menu. It is not acceptable that choices be made days before the actual meal, as residents may not remember what they have selected when service takes place [14,18]. This has been shown to negatively affect residents’ satisfaction with the meal experience [1].

### 1.3. Variety in Meal Choice

In the aged care setting, menus are often cyclic with limited alternative choices [19]. Rotating menus may be used as a strategy for promoting choice by changing the menu often [20]. Positive effects on meal consumption and enjoyment can be achieved through liberalising meals and avoiding restrictions of nutrients, particularly sugar and fat [1], and by providing a variety of meal options for residents on texture modified diets which are integrated into the general menu. The range of foods that can be made available is influenced by both the production and the meal delivery systems. If the production system requires menu forecasting of production quantities in advance, then residents will have to make choices before the day of service. Meal delivery systems such as tray services usually cannot provide the same level of flexibility with respect to choice, as the menu items need to be able to be served onto the specific trays.

### 1.4. Texture Modified Diets

Aged care menu planning must include quality planning for textured modified meals. Quality menu planning includes ensuring that the non-texture modified and texture modified menus, particularly the most texture modified pureed meals, include the same number of options for residents [21,22]. The change in texture may result in foods losing their shape [23]. They are less appealing from a sensory perspective and are often nutritionally dilute through the addition of extra fluid [21,24].

The extent of menu choice available in Australian RACHs is unknown. The expected outcomes from the Australian Aged Care Accreditation Standard 4.8 (Catering, Cleaning and Laundry services) and Standard 2.10 (Nutrition and Hydration) do not provide guidance on the process for achieving adequate/any choice in menu planning. The standards are outcome-based rather than process-oriented [25]. It is practically known that individual homes interpret menu design for themselves and determine the level of choice on the menu within the limits of the production system. Australian Aged Care Accreditation Standard 3.9 (Choice and Decision Making) pertaining to resident choice does not include food choice, or any guidance on an acceptable level of choice in menu planning [25].

The aims of this study were to provide insight into: (i) how menus in residential aged care homes are planned to offer choice to residents; (ii) the options available for alternative choices; and (iii) the quality (*i.e.*, variety) built into menu planning for both the general and texture modified menus in the Australian context.

## 2. Methods

The critical realism framework was used to undertake this work, as it allows for in-depth causal explanation taking into account a wide variety of information from various sources [26]. Three separate studies using different data collection techniques were undertaken and the data from all three studies was interpreted together (*i.e.*, triangulated) to improve confidence in the results. The three studies were conducted over a three-year period (2010–2013). Ethical approval for study one and two was obtained from the University of Queensland Human Research Ethics Committee # HMS09/0212. Study three was approved by the University of Queensland Human Research Ethics Committee #12/1106.

### 2.1. National Menu Survey

The National Menu Survey was designed to ascertain the variety of menu planning practices used to facilitate resident meal choice in Australian RACHs. The survey was mailed to the Hotel Services/Catering Manager or equivalent at 2664 RACHs across Australia. Homes were identified using the computerised Australian Aged Care Database on CDROM (2010) [27]. Informed consent was indicated by RACHs completing and returning the survey within 14 weeks. Reminders were sent through notices in online publications “Aged Care and Community Services” and the “Aging Agenda”. Respondents were asked to include a copy of their menu with their returned surveys.

The survey contained three main sections: (i) general information about the RACH and foodservice system; (ii) menu information and (iii) staffing information. In part one, participants were asked to provide information on the type of home, the location, facility size, care classification, type of foodservice production system (and whether in-house or outsourced), meal delivery system/s, the role classification of the person responsible for managing food services and the level of dietetic involvement in foodservices operations. Respondents were asked to indicate from a set list of possible time frames, when residents had to choose their meals. This ranged from on the day of service to greater than 24 hours prior to service. In part two, participants were asked to indicate the menu cycle length and seasonal menu changes, in addition to the meal components used for breakfast, lunch, evening meal, mid-meals and fluids. There was space for adding the types of texture modified mid-meal snacks provided. This section also included questions about the types of special diets catered for, and how additional foods and supplements (liquid, powdered and pudding type) were used.

Homes were asked if they undertook food fortification menu strategies and to detail the type and frequency of these strategies. To gain a sense of how menus were planned, homes were asked to provide information on how the obtain feedback from residents about the menu, barriers to menu planning and what guides their selection of food items for their menus. Homes were also asked to provide information about portion sizes for the general and texture modified meals, either through standardised recipes or actual portion size in grams. In part three, homes were asked to provide information on resident per day per meal costs, which comprised both labour and food. This data will be presented elsewhere.

### 2.2. Menu Review

A detailed review of the menus provided with the returned surveys was completed for both the texture modified and non-texture modified diets. Menus were analysed for the amount of choice, meal alternatives offered, and instances where choice was included in the menu across the entire day’s menu pattern.

### 2.3. Observational Study

Case study methodology was used to complete detailed observations within the meal environment (*i.e.*, dining room) across 36 RACHs in New South Wales and South Australia. Recruitment of homes continued until data saturation was reached. The Foodservice and Meal Environment Tool (FAMET) was developed to facilitate structured observation, as no complete meal environment tool could be found. A reflective journal was also kept for each site to record other observations, impressions and reflective thoughts. Each home provided a copy of their menu and portion size specifications. A total of 36 aged care meal environments were observed for all three main meals and morning and afternoon tea snacks (a total of 108 meals and 72 snacks). Of those 36, 30 meal environments included residents who had been prescribed texture modified diets.

Homes were selected based on their differences in production systems, location (metropolitan, regional) and private *versus* not-for-profit ownership. The observations used validated tools to record the actual menu served for both texture modified and non-texture modified diets. This was then compared to the published menu obtained from each participating RACH. Photographs were taken to validate the recorded data collection. The timing of meal choice (*i.e.*, same day service up to more than 24 h prior to the meal service) was also noted for each meal observed. All data was coded numerically, de-identified and entered into SPSS (Student version 18, Chicago, IL, USA). For all three studies frequency analysis was undertaken and Pearson Chi-square tests were used to examine statistical differences between proportions, with statistical significance (two-tailed) assessed at the 5% level (*p* < 0.05).

## 3. Results

Table 1 outlines pertinent summary data for the three studies. The response rate for the national menu survey was 274/2664 (10%). Although this was a low response overall, the study sample was representative for location of RACHs as 66% were in metropolitan and 34% in rural areas. This was not statistically different to the proportions for the national average of 57% and 43%, respectively. Around 11% of the homes were Government owned, while 59% were not for profit, 28% were for profit and 2% did not specify. These proportions were also not statistically different to the national average. Of those homes that responded to the survey, 161 (59%) provided a copy of their menu for further analysis in study two.

**Table 1:** Residential aged care home characteristics and foodservice systems across studies.

Residential Aged Care Home Characteristics	Study 1: National Menu Survey *n* (%)	Study 2: Menu Analysis from Survey *n* (%)	Study 3: Observational Study (Meal Environments) *n* (%)
Sample size	274 (10)	161 (59)	36 (NA) *
Total bed numbers in study populations	20808	12126	1104
*Menu cycle length*			
3 weeks or less	18 (6)	6 (4)	0 (0)
4 weeks	210 (77)	135 (84)	29 (81)
More than 4 weeks	46 (17)	18 (12)	7 (19)
*Production system*			
Cook fresh	194 (71)	117 (73)	24 (64)
Cook chill	33 (12)	44 (27)	12 (33)
Cook freeze	1 (1)	0	0
*Meal delivery system*			
Decentralised bulk	111 (41)	86 (53)	15 (42)
Tray	163 (59)	75 (47)	21 (58)
*Timing of choice*			
Point of service or a few hours before meals	124 (45)	NA	2 (6)
24 hours prior to consumption	75 (27)	NA	9 (25)
2 days of more before meal consumption	75 (27)	NA	25 (69)

* 30 meal environments had residents requiring texture modified foods.

### 3.1. General Findings

The most frequent menu cycle throughout RACHs was four weeks in length. The typical production system was cook-fresh, and the most common meal delivery systems were decentralised bulk or tray services.

#### 3.1.1. Choice

Main meals on the menus provided were examined for the types of meals and the extent of choices offered. Table 2 outlines the proportion of menus offering various meal components, e.g., a hot breakfast. It can be seen that 47% of RACHs offered a hot breakfast, and there was one hot option available for consumption in all cases. A number of homes offered cold breakfast cereals or juice as alternatives to the hot breakfast, but not all. For the general menu, around 6% of homes offered alternative options for morning tea, while 36% offered alternatives for lunch. 2% offered alternatives for afternoon tea, while >80% offered alternatives for the evening meal. Around 28% offered a couple of choices for the evening dessert. There was no alternative offered for supper in any home.

Results for the general menu and texture modified diet menus obtained are shown side by side for comparison purposes. Only three (2%) of menus offered a hot breakfast for those on texture-modified diets. Similarly, for all meals apart from the hot entrée, homes offered no choice to residents on texture modified diets. This presents a distinct disparity between the choices available for those on the general menu compared to those with special needs with regard to texture modification. A large number of homes failed to indicate on their written menu how they were integrating texture modifications into their menu. This is an aspect of menu planning which requires further investigation to ascertain how this impacts upon the quality of meals being planned in aged care homes.

**Table 2:** Proportion of menus with specific meal components and the choice available for each meal component (*n* = 161).

Meal Components	Options Available	General Menu Frequency (%) *	Texture Modified Diets Menu Frequency (%) *
Hot breakfast	One choice	76 (47)	3 (2)
Cold breakfast cereals	At least four choices	2 (1)	6 (4)
	Four or more choices	39 (24)	0 (0)
Fruit juice choice	One choice	2 (1)	20 (12)
	Two choices	23 (14)	0 (0)
	Three choices or more	88 (55)	0 (0)
Morning tea	One choice	65 (40)	6 (4)
	Two choices	7 (4)	
	Three choices	3 (2)	
Lunch	One choice	103 (64)	10 (6)
	Two choices	58 (36)	
Lunch vegetables	Three vegetables	105 (65)	6 (4)
	Four vegetables	22 (14)	
Dessert	One choice	141 (88)	NA
	Two choices	17 (11)	
Afternoon	One choice	58 (36)	17 (11)
	Two choices	4 (2)	
Soup	One choice	142 (88)	8 (5)
Hot entrée	One choice	140 (87)	0
	Two choices	10 (6)	17 (11)
Evening Meal of hot entrée, soup salad or sandwich combinations	One choice	16 (10)	NA
	Two choices	62 (39)	
	Three choices	15 (9)	
	Four choices	68 (42)	
Evening dessert	One choice	63 (39)	NA
	Two choices	45 (28)	

* The percentage was calculated with the total number of menus available for analysis as the denominator (*i.e.*, *n* = 161).

#### 3.1.2. Timing of Meal Choice

Choice of meals was not made on the day of service in over half the RACHs. Around 45% of residents were able to choose their meals close to point of service, either at the time or a few hours prior.

### 3.2. Observational Study

#### 3.2.1. Choice

Data obtained through observations in 36 meal environments was mostly consistent with that resulting from the National Menu Survey. Of those 36 meal environments observed, 30 provided data on texture modified diets. No hot breakfasts were provided at any of the meal environments observed. The general menu had many choices for cereal and the texture modified diet only offered porridge or Weetbix™ in some cases, or no choice in other cases. No choice was provided for morning tea, lunch, afternoon tea, evening meal (soup), hot entrée, evening dessert or supper for either residents on the general menu or the texture modified menu. Two choices were offered at over half of the meal environments for the lunch dessert for those on the general menu (56%).

#### 3.2.2. Menu Adherence

For the texture modified menu, morning and afternoon teas were always made up on the day, and were inconsistent with the written menu. In around 30% of observations, different meat was used at lunchtime compared with that indicated on the menu, and in 40% of observations the lunch dessert was different. A different dinner meal or meals, left over from the previous day, were provided to residents on the texture-modified menu in around 20% of observations. It was noted consistently that in any meal environment, the written menu for those on texture modified diets was often not followed, meals were repeated on the same day, or repeated from previous days. The menu choice for this group was therefore decreased and the quality of the menu choice was poorer than for those on the general menu. A large proportion of RACHs in the observational study planned the textured modified menu on the day of service and were not using the prepared menu, resulting in the use of left overs and repeated meals. An interesting observation was that some homes restricted food items such as jelly and ice cream for residents on a pureed diet even though most residents were not on thickened fluids and the restriction was unnecessary, further reducing the quality of menu items offered.

## 4. Discussion

The primary outcome of this study was the collation of evidence from menus from RACHs indicating a low level of choice of meals for residents on both general and texture modified diets, and significantly less choice for the latter group. This is contradictory to the quality care principles that state that all services should be of the same quality to meet the needs of all [28]. This is the first study to provide detailed data around these issues and to systematically examine menu planning processes in RACHs across Australia. It was national in its focus and used multiple strategies to increase confidence in the results obtained.

Internationally, choice has been reported to be one of the primary reasons for improved food intake in RACHs [29,30]. Choice also improves residents’ quality of life [31] improves resident autonomy and engagement and increases food intake [32]. As such, it is an important consideration of the quality of service. Further investigation through the observational study revealed the majority of homes did not adhere to their written menus for either general or texture modified diets 20% to 40% of the time. Consequently, this resulted in options made up on the day, repeated meals or the use of leftovers from the previous day. Overall, our results revealed menu planning practice in the RACHs included in the study was not optimised, requiring significant improvement and standard guidelines to assist this process.

Although many homes in this study had cook fresh production systems with either a bulk or tray meal delivery system, there was little evidence of point of service choice, meaning the production system needed to forecast many days before to fill menu requirements. The homes in the study relied heavily on tray meal delivery systems (59%) that are known to reduce flexibility, resulting in residents having to pre-order for these to be set up correctly. In addition, other supporting systems for the menu such as tray meal delivery services limit the choice to residents and can further hinder optimum delivery of the menu. While residents may be able to choose certain parts of their meals, it is not at the point of service, and frequently, cognitive decline can lead to forgetting the choices made, leading to reduced satisfaction with meals [1].

The Australian Aged Care Standard 3.9 is the expected outcome on choice, but there are no foodservice indicators translating this choice into menu options or guidelines for menu planning. Similarly, the Australian Aged Care Standards make no allowances for any choice in texture modified diets. The expected outcomes only make reference to texture modification being made available, and preferences being taken into consideration. How this is supposed to translate into practice is not discussed or suggested. The evidence provided by our studies suggests that this rarely occurs, and it was clear that adherence to residents’ preferences was not achieved. The core issue with the Australian Aged Care Standards and expected outcomes is that they are outcome based rather than process oriented and are open to interpretation by individual RACHs. Linking the meal environment to standards and guidelines to support and provide the structure in which aged care homes should operate, especially in terms of quality of care of residents on a texture modified meal, is imperative. There is evidence indicating that the more restricted food choice becomes, the greater the threat to the resident’s nutritional status [33]. Serving residents the same meal or leftovers would reduce both the choice and variety of foods, leading to risk of poorer nutritional intake. Having no alternative menu choices and receiving left overs or the same meal twice in one day could not be considered a quality service.

With the menu system issues identified here and the lack of menu design statements from the Australian Aged Care Standards, it is easy to understand why homes do not have a stronger menu planning focus when it comes to choice options for residents. The Australian Aged Care Standards are significantly more general than those utilised internationally. For example, the Ministry of Health, Canada, includes Standard 71 Menu planning (c) “alternative choices of entrees, meats, vegetables and desserts at lunch and dinner”, and this is required for both the general and texture modified menu plan [32]. Australian Aged Care Standard 4.8 provides little guidance about the number of alternative choices of foods required at each meal. However, the Canadian standards mandate that residents must be offered two choices at meal times [34,35]. There is no such regulation in Australia, as evidenced by the results obtained in this study.

### Limitations

The written information on menus collected in this study was poor in detail and therefore, it was not easy to determine if residents had a choice as to meal make up, for example the type of sandwich, or just received a mixed sandwich. This case study model may lead to reduced objectivity as only one observer was used; however, this was reduced by only spending one day in each meal environment. The overall sample size was small, but there was good representation of a range of facility types. Despite this, it is unclear if all findings are generalisable to the broader range of aged care homes, as it is possible that facilities with higher compliance with the guidelines were more likely to respond, introducing the potential for non-response bias. Financial implications are an important consideration in the strengthening of implementation of quality standards and choice; however, there is no data on this. Further work building on the results of this study is therefore urgently needed.

## 5. Conclusions

This study highlights the need for changes to the Australian Aged Care Standards to protect residents’ rights to have meal choice and ensure equality in menu planning for all. Regardless of diet type, all residents in homes should have access to the same quality of service provision. Residential aged care in Australia has seen a shift over the past 10 years to caring for residents who are older and frailer with lower cognitive function [36]. The ability to plan and design menus requires feedback from residents who are increasingly unable to participate in this process. Having a well-planned menu is no longer enough. The dining room and a successful eating experience are essential to ensure the residents are meeting their nutritional targets. Examining how this interacts with the menu planning process should be the focus of future work. Regulation and monitoring of the Australian Aged Care Accreditation Standards needs to be strengthened to mandate improvement of the choice and variety offered to residents, particularly those on texture modified diets. Further research on how menu choice and a lack of variety in meals affects the quality of life residents is needed in this context, but current evidence suggests the effect would be detrimental and undermine resident autonomy and nutritional status, thereby placing aged care residents at significant risk.
